# Food colorant brilliant blue causes persistent functional and structural changes in an in vitro simplified microbiota model system

**DOI:** 10.1093/ismeco/ycaf050

**Published:** 2025-03-22

**Authors:** Victor Castañeda-Monsalve, Sven-Bastiaan Haange, Laura-Fabienne Fröhlich, Qiuguo Fu, Ulrike Rolle-Kampczyk, Martin von Bergen, Nico Jehmlich

**Affiliations:** Department of Molecular Toxicology, Helmholtz Centre for Environmental Research – UFZ GmbH, 04318 Leipzig, Germany; Department of Molecular Toxicology, Helmholtz Centre for Environmental Research – UFZ GmbH, 04318 Leipzig, Germany; Department of Environmental Analytical Chemistry, Helmholtz Centre for Environmental Research – UFZ GmbH, 04318 Leipzig, Germany; Department of Environmental Analytical Chemistry, Helmholtz Centre for Environmental Research – UFZ GmbH, 04318 Leipzig, Germany; Department of Molecular Toxicology, Helmholtz Centre for Environmental Research – UFZ GmbH, 04318 Leipzig, Germany; Department of Molecular Toxicology, Helmholtz Centre for Environmental Research – UFZ GmbH, 04318 Leipzig, Germany; Institute of Biochemistry, Faculty of Biosciences, Pharmacy and Psychology, University of Leipzig, 04103 Leipzig, Germany; German Centre for Integrative Biodiversity Research (iDiv) Halle-Jena-Leipzig, 04103 Leipzig, Germany; Department of Molecular Toxicology, Helmholtz Centre for Environmental Research – UFZ GmbH, 04318 Leipzig, Germany

**Keywords:** SIHUMIx, metaproteomics, metabolomics, brilliant blue, food colorants, human gut microbiota

## Abstract

The human gut microbiota plays a vital role in maintaining host health by acting as a barrier against pathogens, supporting the immune system, and metabolizing complex carbon sources into beneficial compounds such as short-chain fatty acids. Brilliant blue E-133 (BB), is a common food dye that is not absorbed or metabolized by the body, leading to substantial exposure of the gut microbiota. Despite this, its effects on the microbiota are not well-documented. In this study, we cultivated the Simplified Human Microbiota Model (SIHUMIx) in a three-stage in vitro approach (stabilization, exposure, and recovery). Using metaproteomic and metabolomic approaches, we observed significant shifts in microbial composition, including an increase in the relative abundance of *Bacteroides thetaiotaomicron* and a decrease in beneficial species such as *Bifidobacterium longum* and *Clostridium butyricum*. We observed lower protein abundance in energy metabolism, metabolic end products, and particularly lactate and butyrate. Disturbance in key metabolic pathways related to energy production, stress response, and amino acid metabolism were also observed, with some pathways affected independently of bacterial abundance. These functional changes persisted during the recovery phase, indicating that the microbiota did not fully return to its pre-exposure state. Our findings suggest that BB has a lasting impact on gut microbiota structure and function, raising concerns about its widespread use in the food industry. This study underscores the need for further research into the long-term effects of food colorants on the gut microbiota and their potential health implications.

## Graphical Abstract

**Graphical Abstract:**
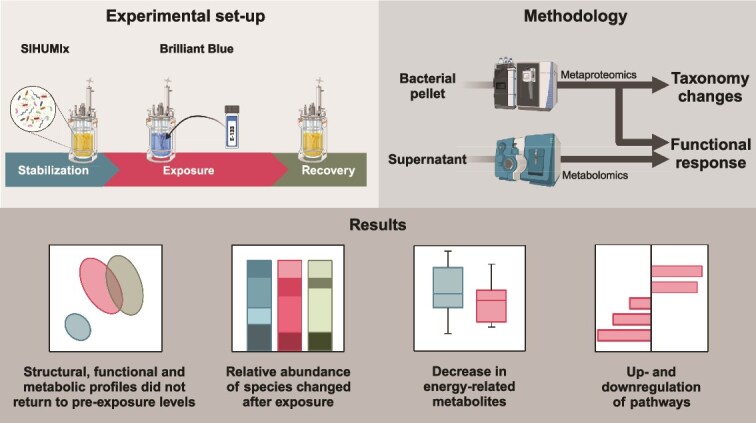

## Introduction

The human gut microbiota is a complex and dynamic community of microorganisms that exist in symbiosis with the host, playing a crucial role in overall health. This microbial community resides in the digestive tract, protecting the host against pathogens [1, 2]. Additionally, it metabolizes not only complex carbon sources but also proteins, vitamins, and xenobiotics to produce beneficial substances, such as short-chain fatty acids (SCFAs), which serve as nutrients for colonocytes, enhance intestinal physiology, and modulate the immune system. [3–5].

Several external factors can influence the gut microbiota, including age, geographic location, diet, and the intake of substances such as food additives and colorants, medications, supplements, and residues from agro-industrial products, also known as xenobiotics [6]. When ingested orally, xenobiotics can interact with the microbiota, leading to transformation into active forms, inactivation, conversion into toxic forms, or no transformation at all [5, 7]. These interactions can stress the microbiota, altering its structure and function, and therefore changing the profile of metabolites supplied to the host and therefore potentially posing a health risk [8].

Food colorants are xenobiotics commonly added to enhance the appearance of processed products and increase their appeal to consumers. The effects of synthetic colorants on the gut microbiota remain largely unexplored, with most research focusing on specific dye groups, such as titanium dioxide (TiO₂) and azo dyes. Studies have shown that these compounds can promote dysbiosis by altering the Firmicutes-to-Proteobacteria ratio [9, 10], reducing the abundance of beneficial species [11], and inducing gut inflammation [12].

Brilliant blue (BB) is a synthetic dye widely used in alcoholic and non-alcoholic beverages, breakfast cereals, candies, baked goods, and frozen desserts [13]. BB is neither absorbed nor metabolized by the body, with approximately 96% of the ingested amount excreted in faeces [14, 15], suggesting significant interaction between BB and the intestinal microbiota. Studies on the effects of BB on intestinal microbiota are very limited, and existing research has mainly focused on single-strain experiments [16, 17].

In a previous study, we investigated the effect of BB on a simplified human gut microbiota model (SIHUMIx) for 24 hours [18]. The SIHUMIx model comprises eight well-characterized bacterial species that have been genetically and proteomically defined, representing the major phyla commonly found in the human intestinal microbiota. The results revealed notable impacts on the microbial community, including disruptions to its composition, reduced production of several SCFAs, and interference with key metabolic pathways [18]. Given the critical role of the gut microbiota in human health, the widespread use of BB, and its significant interaction with the intestinal microbiota, further investigation is necessary. Specifically, long-term exposure studies are essential to understand how this colorant influence microbial communities and their metabolic functions.

To further explore the interactions between BB and gut microbiota, we conducted a study using the SIHUMIx model, incorporating prolonged 7-day in vitro cultivation followed by a 4-day recovery phase. We utilized metaproteomics to analyse changes in species’ relative abundance and metabolic pathways. Metabolomic techniques were employed to measure SCFA concentrations and profile untargeted metabolomics throughout the cultivation stages. This approach aims to elucidate structural and functional changes within the microbiota and assess its recovery capabilities post-dye removal, providing a comprehensive overview of how readily accessible chemicals impact a simplified gut microbiota model.

## Materials and methods

### Simplified human intestinal microbiota model – SIHUMIx

We used the extended simplified human intestinal microbiota (SIHUMIx) [19], which is composed by a total of eight species which represent the functional core of the human microbiome: *Anaerostipes caccae* (DSMZ 14662), *Bacteroides thetaiotaomicron* (DSMZ 2079), *Bifidobacterium longum* (NCC 2705), *Blautia producta* (DSMZ 2950), *Clostridium butyricum* (DSMZ 10702), *Thomasclavelia ramosa* (DSMZ 1402), *Escherichia coli* K-12 (MG1655), and *Lactiplantibacillus plantarum* (DSMZ 20174). Considerations for its use are detailed in the supplementary methods.

### *In vitro* bioreactor cultivation

**Figure 1:**
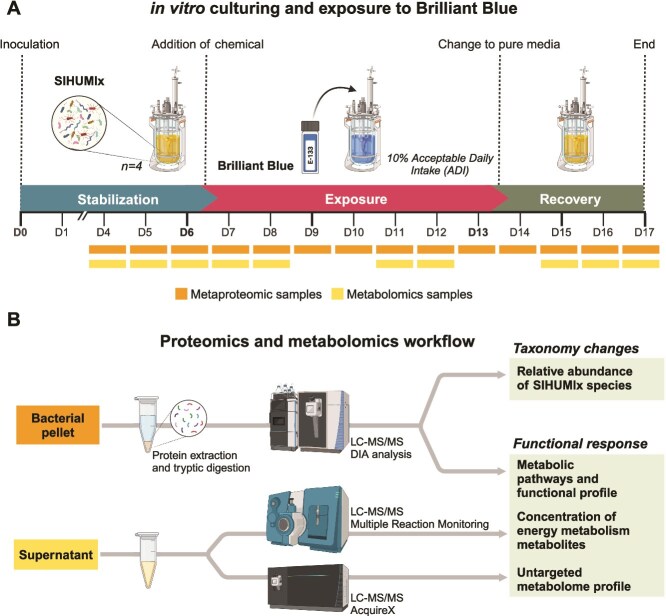
**Experimental design.** We evaluated the influence of brilliant blue (BB) on the simplified human microbiota model (SIHUMIx) using a continuous bioreactor cultivation system. (**A**) after inoculation and a six-day stabilization period, BB was introduced at a concentration of 0.168 mg/ml (10% ADI) and maintained for 7 days. On Day 13, a recovery phase began, during which the chemical was washed out. (**B**) Starting from day four, daily samples were taken for metaproteomic and metabolomic analysis. Created with BioRender.com.

The stability of SIHUMIx in bioreactors has been previously established. Studies using the same cultivation conditions as our study found that SIHUMIx reaches a steady state by day four. Various phenotypic characteristics, such as structural composition, relative species abundances, SCFA production, flow cytometry fingerprints, intact protein profiling, and t-RFLP, remain stable up until Day 21 [20–22]. The experimental design and overall workflow in this study is illustrated in Fig. 1.

Due to the previous considerations, our experiment was designed to compare SIHUMIx within the same reactors across different stages. We use the late stabilization stage, from Day 4, as a reference point for a stable community. This approach strengthens statistical power by increasing the number of bioreactors and avoids the inherent variability that comes from comparing different sets of bioreactors.

Prior to inoculation, each strain was thawed in 9 ml of Brain Heart Infusion (BHI) supplemented with L-cysteine hydrochloride [0.5 g/], vitamin K – Hemin solution [10 ml/L] (Becton Dickinson, Heidelberg, Germany) and yeast extract [5gr/L], and incubated at 175 rpm, 37°C, under anaerobic conditions. Overnight cultures were counted, and 1×10^9^ cells per strain were used for inoculum preparation, resulting in a total of 8 × 10^9^ cells for each bioreactor. On Day 0, four 250 ml Multifors 2 bioreactors (Infors, Switzerland) filled with sterile Complex Intestinal Media (CIM) (Table S1) were inoculated with SIHUMIx in batch mode. The bioreactors were maintained at 37°C with stirring at 200 rpm. Anaerobic conditions were ensured through the constant injection of N₂, and the pH was controlled at 6.5 to replicate conditions typical of the distal colon. After 24 hours post-inoculation, the system was switched to continuous mode with a media exchange rate of 24 hours until the end of the experiment. The previous conditions of pH, temperature, agitation and anaerobic conditions remained the same.

After day six, BB was injected into the bioreactors to achieve a concentration of 10% of the Acceptable Daily Intake (ADI) situated at 6 mg/kg bw/day [23]. We assumed each bioreactor contained approximately 10% of the bacterial cells compared to the 2 × 10^13^ Colony Forming Units found in the daily faecal matter expelled by an average 70 kg adult [24], resulting in a final concentration of 0.168 mg/ml of BB. To maintain a stable concentration during the 6-day exposure period until Day 13, the feed media contained BB at the same concentration.

To assess the recovery to its pre-exposure state, the concentration was gradually reduced. At the end of Day 13, feed was switched back to medium without xenobiotic. The recovery stage lasted four days, extending the total cultivation period to 17 days. This duration prevents biofilm formation, which typically occurs in our system by Day 21. Daily samples were taken from Day 4 onwards. Four aliquots of 2 ml each, were taken from each bioreactor and centrifuged at 5000 g for 10 minutes, pellets and supernatants were separated and preserved at −80°C for subsequent analysis.

### Sample preparation for metaproteomic analysis

Pellet lysis and protein extraction procedures adhered to the protocol outlined in Castañeda-Monsalve et al., 2024. The resultant supernatant, underwent determination of protein concentration utilizing the Pierce 660 nm Protein Assay (Thermo Fisher Scientific, Waltham, MA, USA).

For the protein clean-up and digestion steps, a modified iteration of Single-Pot Solid-Phase-enhanced Sample Preparation (SP3) was employed [25], the detailed protocol is described in the supplementary methods. Resulting peptides were transferred into LC–MS vials and stored at −80°C until LC–MS/MS measurements.

### LC–MS/MS measurements

NanoLC–MS measurement measurements were conducted using Vanquish Neo nanoHPLC system (Thermo Fisher Scientific), coupled to an Orbitrap Exploris™ 480 mass spectrometer (Thermo Fisher Scientific) operating in data-independent acquisition mode (DIA). Detailed liquid chromatography and mass spectrometry parameters can be found in the supplementary methods.

### Spectral library building for DIA data analysis

To construct the spectral library, we utilized 490 non-fractionated data-dependent acquisition (DDA) files containing measurements of SIHUMIx conducted in our laboratory using Spectronaut™ Pulsar (v18.6.231227.55695, Biognosys AG, Schlieren, Switzerland). Protein inference was achieved through protein sequence databases specific to the eight SIHUMIx species, obtained from UniProt Proteomes (www.uniprot.org): *A. caccae* (3743 entries), *B. thetaiotaomicron* (4782 entries), *B. longum* (1725 entries), *B. producta* (5372 entries), *C. butyricum* (4015 entries), *T. ramosa* (3166 entries), *E. coli* K-12 (4448 entries), and *L. plantarum* (3087 entries). The resulting library comprises 1 273 751 fragments, 212 981 precursors, 12 318 unique protein groups, and 174 109 peptides.

### Metaproteome DIA data analysis

The processing of mass spectrometric data was carried out using Spectronaut™ (Biognosys AG). DIA raw files were analysed using the previously generated SIHUMIx spectral library with default settings in the Biognosys generic format. The protein sequence databases utilized were consistent with those employed in constructing the spectral library. Additionally, the prediction of retention time was performed using iRT.

To identify the functions of proteins, the sequences of the discovered proteins were uploaded to the Kyoto Encyclopaedia of Genes and Genomes (KEGG) website (www.genome.jp/kegg/). Using GhostKoala, KEGG orthologs were annotated to the proteins. The relative abundances of each SIHUMIx species were quantified by estimating their protein biomass, enabled by label-free quantification methods. The summed intensity of all proteins associated with each microorganism was calculated, and its proportion relative to the total intensity of all bacterial proteins in each sample was used to determine the relative abundance of each bacterium across the experimental phases. For further analysis, only pathways containing at least five proteins and having a minimum total coverage of 15% were selected.

### Analysis of SCFAs

The targeted quantification of SCFAs followed a previously established protocol [26]. Please refer to the Supplementary Information document sample preparation and metabolite quantification details.

### Untargeted metabolomics

Untargeted metabolomics analyses were conducted using high-performance liquid chromatography (HPLC) coupled to an Orbitrap IQ-X mass spectrometer (Thermo Fisher Scientific), operated in AcquireX mode. For detailed sample preparation, chromatography parameters and metabolites identification and quantification please refer to the supplementary methods.

### Measurement of brilliant blue

For the quantitative analysis of BB and its possible transformation products, daily supernatant samples were analysed using ultraperformance liquid chromatography coupled with time-of-flight mass spectrometry (UPLC-TOF-MS). The analytical methods were based on previously established protocols [27], and quantification was performed in a manner similar to that used in prior studies [28–30]. Detailed descriptions of the LC–MS parameters and the complete analytical procedure are provided in the supplementary methods.

### Statistics

All statistical analyses were conducted using R. Differences between cultivation stages were determined by the Kruskal-Wallis test (KW-test), followed by pairwise comparisons using Dunn’s test. Taxonomic, metabolomic, and functional profiles were observed using principal component analysis (PCA), with subsequent significance testing via permutational multivariate analysis of variance (PERMANOVA) using the *adonis2* function from the *vegan* package. Functional adaptation of the bacterial strains during the exposure phase were determined with the Spearman correlation coefficient using the *rcorr* function in the Hmisc package. For multiple comparisons involving more than 20 independent tests, P-values were adjusted according to the Benjamini-Hochberg method.

## Results

### Dye concentration across incubation stages

**Figure 2:**
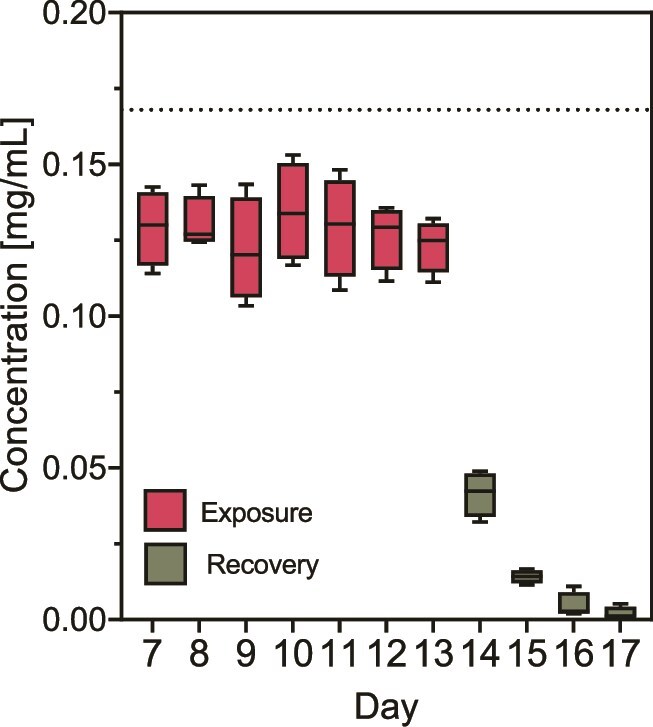
**Levels of brilliant blue across the experiment.** The concentration of BB across the stages of exposure and recovery was measured in the culture supernatant and represented in boxplots. Stabilization phase samples all had a concentration of 0 mg/ml (data not shown). The dotted line indicates the theoretical concentration (0.168 mg/ml). Error bars represent the standard deviation of biological replicates (n = 4).

To realistically represent the permitted exposure levels to BB, the experiment was designed so that during the exposure phase, the calculated concentration was 0.168 mg/ml of BB. Daily measurements of the concentration in the supernatant revealed that the average concentration of BB in the bioreactors was 0.127 ± 0.0125 mg/ml (Fig. 2). Subsequently, during the 4-day recovery period, the concentration gradually decreased to 0.002 ± 0.002 mg/ml. No transformation products were found during exposure and recovery stages.

The concentration found in the supernatant during the exposure phase corresponds to approximately 73% of the calculated concentration. We estimate that the remaining dye may have been absorbed or adsorbed by bacterial cells, adhered to the surfaces of the bioreactors, or bound to protein components of the culture medium. Studies to determine the location of the remaining dye were not conducted.

**Figure 3:**
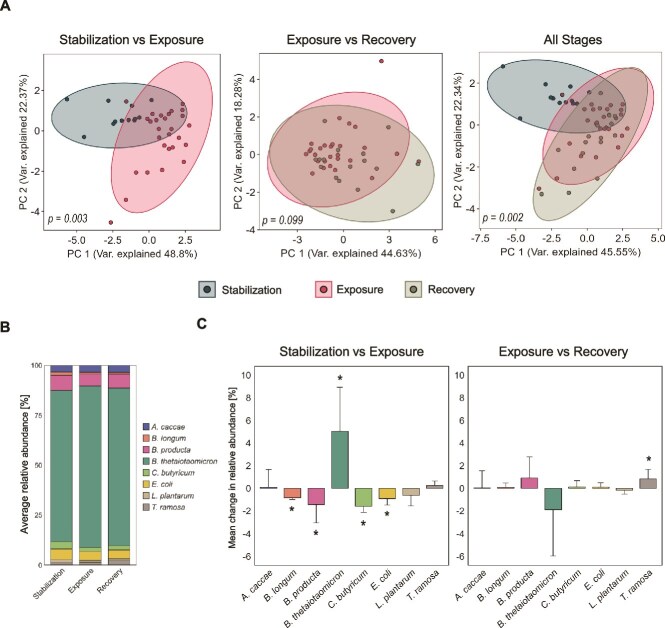
**Impact of brilliant blue on the composition of SIHUMIx.** Analysis of the structure of SIHUMIx during the stages of the experiment. (**A**) PCA of species’ relative abundance, comparing stabilization to exposure, exposure to recovery, and all stages combined, significance calculated by PERMANOVA. (**C**) Mean relative abundance of species during the stages of cultivation. (**B**) Change in the relative abundance of each SIHUMIx species from stabilization to exposure and from exposure to recovery. Error bars represent the standard deviation, and asterisks indicate significance calculated by the Kruskal-Wallis test (*P* < .05).

### Detected protein numbers

To investigate the functional and structural alterations in a simplified human microbiota model exposed to BB, we employed a metaproteomic analysis. Across all examined samples, we identified a total 5387 proteins assigned to 4938 protein groups. The average number of protein groups per sample was 4371, with an interquartile range of 4237-4546.

### Exposure to brilliant blue affected SIHUMIx structure

After analysing the abundance profile of SIHUMIx species exposed to BB for seven days, followed by a recovery period, we observed significant changes in the taxonomic structure. PCA revealed differences between the stabilization and exposure phases (PERMANOVA, *P* = .003). However, no significant differences were detected between the exposure and recovery phases (PERMANOVA, *P* = .099) (Fig. 3A), while the stabilization and recovery phases remained different, suggesting that the community did not return to a state similar to stabilization (PERMANOVA, *P* = .012) (Fig. S1A).

All SIHUMIx species were detected throughout the experiment (Fig. 3B). During the exposure to BB, *B. thetaiotaomicron* significantly increased in relative abundance (KW-test *P* = .0003), whereas *B. longum, B. producta, C. butyricum*, and *E. coli* decreased (KW-test *P* < .05). Despite the overall taxonomic profile of SIHUMIx showing no significant differences between the exposure and recovery phases (*P* = .099), *T. ramosa*, whose abundance was unaffected during the exposure phase, significantly increased during the recovery stage (KW-test *P* = .0001) (Fig. 3C).

### Brilliant blue exposure reduced lactate and propionate concentrations and pathways involved in their production

The production of SCFAs is a marker of bacterial energy metabolism. Alterations in these metabolites, and the proteins involved in their metabolic pathways, can indicate potential stress effects that BB might have on SIHUMIx. This study detected nine SCFAs and lactate in the supernatants on the sampled days. We compared the concentration of each metabolite during the stabilization, exposure, and recovery phases (Fig. S2).

**Figure 4:**
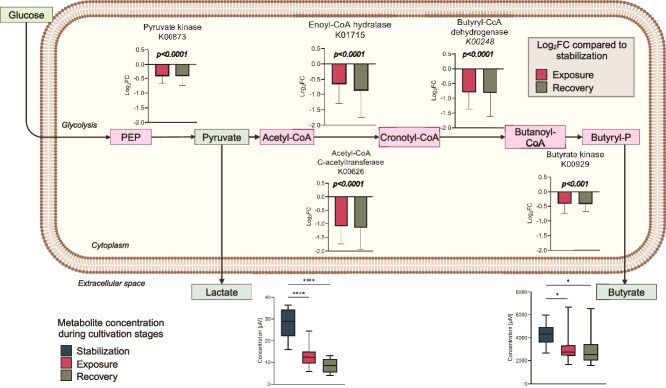
**Brilliant blue affected energy-metabolism pathways and its products.** Snapshot of the effects of BB on pyruvate metabolism and butyrate metabolism pathways in the representation of a general bacterial cytoplasm, represented by the Log_2_FC of affected proteins detected during the exposure phase compared to the stabilization phase. In the extracellular space, concentrations of the metabolites lactate and butyrate are shown during the stabilization and exposure stages. Error bars represent the standard deviation, and significance was calculated by the Kruskal-Wallis test (*P* < .05). Created with BioRender.com

Among the primary metabolites (acetate, butyrate, propionate, and lactate), significant differences were observed only in the concentrations of butyrate (KW-test *P* = .0078) and lactate (KW-test *P* < .0001) between the stabilization and exposure stages (Fig. 4). Additionally, the lactate concentration significantly decreased further during the recovery stage (KW-test *P* < .05) (Fig. S2).

To understand the impact of BB on the molecular processes involved in butyrate and lactate production, we analysed the intensities of the metabolic pathways KEGG 00620 (pyruvate metabolism) and KEGG 00650 (butyrate metabolism). Comparing the summed intensity of protein groups corresponding to each metabolic pathway between the cultivation phases, we found a significant reduction in both pathways during the exposure stage compared to the stabilization phase (KW-test *P* < .001). The intensity of proteins in the pyruvate metabolism pathway continued to slightly decrease during the recovery phase (KW-test *P* < .05) (Fig. S3).

Further analysis revealed that within the pyruvate metabolism pathway, the enzyme pyruvate kinase (Log_2_FC = −0.4, KW-test *P* < .0001) was significantly reduced during exposure. Similarly, in the butyrate metabolism pathway, enoyl-CoA hydratase (Log_2_FC = −0.7, KW-test *P* < .0001), acetyl-CoA C-acetyl transferase (Log_2_FC = −1, KW-test *P* < .0001), butyryl-CoA dehydrogenase (Log_2_FC = −0.8, KW-test *P* < .0001), and butyrate kinase (Log_2_FC = −0.4, KW-test *P* < .001) were also significantly reduced during exposure to BB (Fig. 4).

### Functionality of SIHUMIx shifted after exposure to brilliant blue

**Figure 5:**
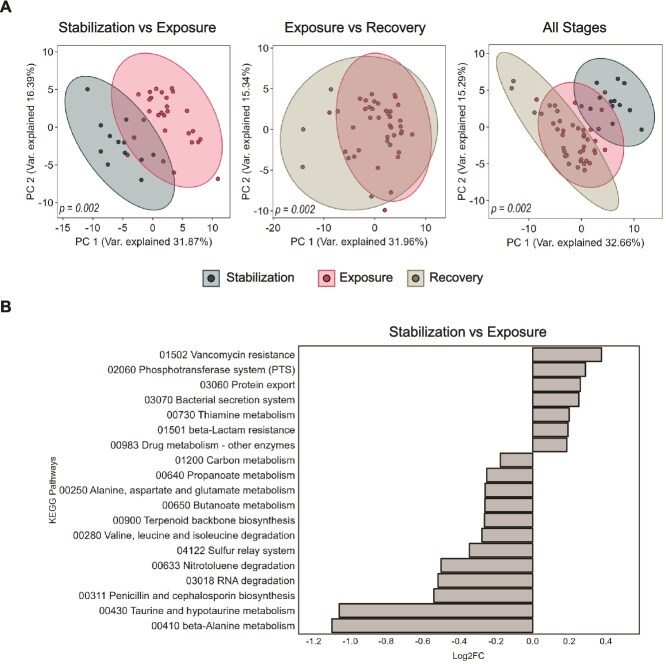
**Brilliant blue changed SIHUMIx functional profiles and pathways.** Analysis of the functional profiles of SIHUMIx after exposure to BB was conducted. (**A**) A principal component analysis (PCA) of pathways’ relative abundance compared the stabilization phase to the exposure phase, the exposure phase to the recovery phase, and all stages combined. Significance was determined using PERMANOVA. (**B**) Pathways of interest were identified by comparing the stabilization and exposure phases. These pathways were filtered based on a threshold of Log_2_FC = ±0.175 and statistical significance, as calculated by the Kruskal-Wallis test (*P* < .05).

To understand the metabolic changes in SIHUMIx caused by BB, analyses of proteins grouped into metabolic pathways were conducted. Out of 4938 protein groups identified in the samples, 3525 were assigned to KEGG orthologues and 79 KEGG metabolic pathways. A metabolic pathway was considered if it had at least 15% functional coverage and a minimum of five assigned proteins. Using PCA to determine clusters and differences in functional profiles between phases based on the relative abundance of each metabolic pathway, significant differences were found between the stabilization, exposure, and recovery phases (PERMANOVA, *P* = .002) (Fig. 5A and Fig. S1B). To filter the most relevant metabolic pathways based on the magnitude of effect, criteria included a KW-test p-value <0.05 and a log_2_FC ±0.175, focusing on comparisons between the exposure vs. stabilization and stabilization vs. exposure phases.

Comparing the exposure and stabilization phases (PERMANOVA, *P* = .002), 19 significant metabolic pathways were identified (KW-Test p-value<0.05) (Fig. 5B). Increased pathways included those involved in compound expulsion from the cell, such as Protein export (Log_2_FC = 0.26) and the Bacterial secretion system (Log_2_FC = 0.25), antibiotic resistance like Vancomycin resistance (Log_2_FC = 0.38) and beta-lactam resistance (Log_2_FC = 0.2), and xenobiotic metabolism such as Drug metabolism (Log_2_FC = 0.19). Conversely, during exposure to BB, decreased pathways included those involved in energy metabolism, such as carbon metabolism (Log_2_FC = −0.176), and propionate (Log_2_FC = −0.25) and butanoate metabolism (Log_2_FC = −0.26), as well as amino acid metabolism pathways like alanine, aspartate, and glutamate metabolism (Log_2_FC = −0.26), taurine and hypotaurine metabolism (Log_2_FC = −1.06), beta-alanine metabolism (Log_2_FC = −1.1), as well as valine, leucine, and isoleucine degradation (Log_2_FC = −0.27). During the recovery stage compared to the exposure stage (PERMANOVA, *P* = .002) (Fig. 5A), eight metabolic pathways were affected, with amino acid metabolism pathways continuing to decrease, while the phosphotransferase system, responsible for sugar incorporation into cells, increased in both phases (Log_2_FC > 0.29) (Fig. S4).

**Figure 6:**
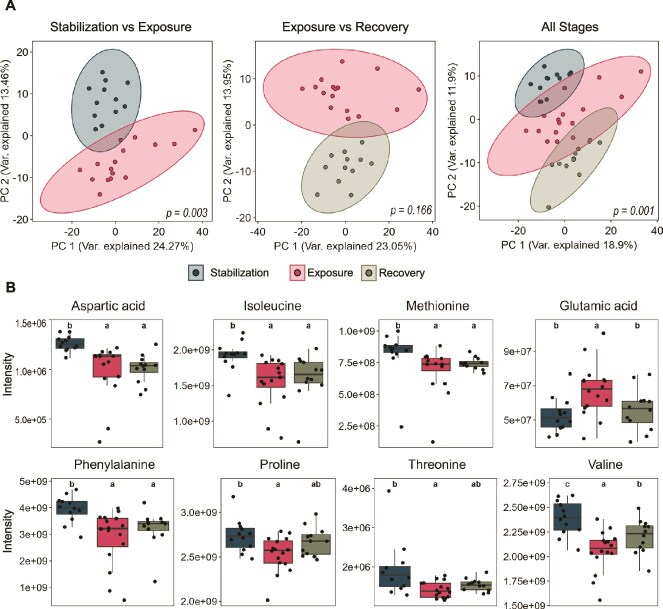
**Analysis of the untargeted metabolomics results after exposure to brilliant blue.** (**A**) Principal component analysis (PCA) of detected metabolite intensities, comparing stabilization to exposure, exposure to recovery, and all stages combined. Significance was calculated by PERMANOVA. (**B**) Intensities of eight amino acids detected during the stages of the experiment. Letters above plots represent significant differences in the post-hoc test, calculated by the Kruskal-Wallis and Dunn’s tests (*P* < .05), points represent samples taken from the four bioreactors during each cultivation phase.

### Brilliant blue exposure shifted global metabolite profile of SIHUMIx

After the untargeted search for metabolites, 556 were detected across samples during the three stages. Metabolite profiles were compared across these stages, similar to the comparison done for abundance and functional profiles. Significant differences were observed between the metabolite profiles of the stabilization and exposure stages (PERMANOVA, *P* = .003). However, no significant differences were found when comparing the exposure and recovery stages (PERMANOVA, *P* = .166) (Fig. 6A), showing trends similar to those observed in the abundance profiles, where stabilization and recovery phases were significantly different (PERMANOVA, *P* = .002) (Fig. S1C).

Noting that metabolic pathways related to amino acid metabolism were negatively affected during the exposure stage, we analysed amino acid levels in the untargeted metabolomics results. 15 of the 20 essential amino acids were detected, except for asparagine, cysteine, glutamine, glycine, and leucine. Among the detected amino acids, aspartic acid, isoleucine, methionine, phenylalanine, proline, threonine, and valine were significantly reduced after SIHUMIx was exposed to BB (KW-test *P* < .05) (Fig. 6B). Although glutamic acid was also significantly affected (KW-test *P* < .05), its levels increased during the exposure stage.

### Temporal responses to SIHUMIx species’ affected pathways

To identify temporal adaptation trends during the days of exposure, a correlation analysis was conducted for the species that were significantly affected. Specifically, five species that showed significant changes during the exposure phase were analysed: *B. thetaiotaomicron, B. longum, B. producta, C. butyricum*, and *E. coli.* The proteomes of these species were examined, and pathways that were among the top 19 showing significant changes in the functional analysis (Fig. 5B) were retained. Notably, *B. longum* did not contribute to any of these pathways. For the remaining four species, the relative abundance of each metabolic pathway was correlated with the days of the exposure phase (Days 7 to 13) and contrasted with the species’ relative abundance during this specific stage. This method ensured that variations in abundances of pathways within the species were unaffected by their overall abundance in the sample. Only correlations with a *P*_adj_ < .05 and a Spearman correlation coefficient of ±0.55 were considered significant.

**Figure 7:**
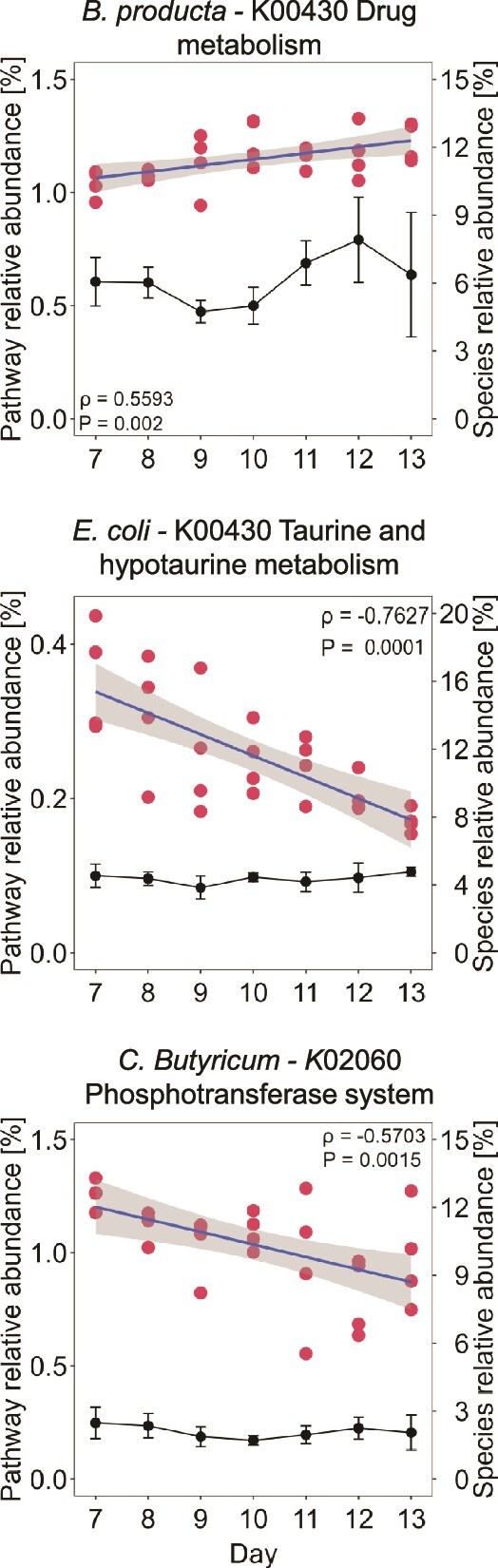
**Exposure to brilliant blue modulates metabolism of affected SIHUMIx species.** Above: Spearman’s correlation analysis was performed to examine the relationship between the relative abundance of selected metabolic pathways and the days of the experiment during the stabilization and exposure phases. Below: Species’ relative abundance during the exposure phase. Only species significantly affected during exposure and contributing to altered pathways, with Spearman correlation coefficients of ±0.55, were considered. p-values were adjusted using the Benjamini-Hochberg method to correct for multiple testing.

In total, three significant correlations between species-specific identified pathways and day of the exposure were found (Fig. 7). During the exposure phase, for *B. producta*, a positive correlation with Drug metabolism (*P* = .55, *P*_adj_ = .002) was identified. For *E. coli*, Taurine and hypotaurine metabolism (*P* = −.76, *P*_adj_ = .0001) showed significant correlation. For *C. butyricum*, Phosphotransferase system showed a significant correlation (*P* = −.57, *P*_adj_ = .0015). No pathways associated with *B. thetaiotaomicron* showed significant correlations.

## Discussion

### Effects on SIHUMIx structure

After exposure, changes were observed in five bacterial species: *B. thetaiotaomicron* increased significantly, while the relative abundances of *B. longum*, *B. producta, C. butyricum*, and *E. coli* decreased. The effect of BB on the microbiota is not well understood, with limited studies investigating its antimicrobial properties mainly in isolated cultures. At the single strain level, significant reductions in optical density were observed at concentrations ranging from 0.03 to 0.24 mg/ml [16, 17], which includes the theoretical concentration used in this experiment (0.168 mg/ml). In our previous study, where SIHUMIx was acutely exposed to BB (Fig. S5), only the abundance of *C. butyricum* was significantly reduced, while the other strains showed no notable changes. The higher number of species affected in this experiment could be due to the constant pressure exerted by the colorant during the seven-day exposure, compared to the single-timepoint experiments reported in the literature.

Each species affected during exposure phase has been reported as a key player in human well-being, and their reduction could threaten gut balance and health. Notably, SCFAs are produced *by C. butyricum*, a butyrate producer [31], *B. producta*, an acetate producer [32], and *E. coli*, which also produces acetate, lactate, and vitamins K2 and B2 [33, 34]. Additionally, these species modulate and protect the intestinal environment: *C. butyricum* supports intestinal homeostasis [35], *B. longum* promotes epithelial function and reduces inflammation [36, 37], *B. producta* stimulates mucosal growth [32], and *E. coli* defends against pathogens [33]. During the exposure phase, we observed an increase in *B. thetaiotaomicron*, an increase in bacteria from the genus Bacteroides is typically associated with good epithelial health; by consuming mucins in the mucus, they promote the rejuvenation of the layers [38]. However, excessive thinning of the mucus layer could allow pathogens to cross the barriers and move to extraintestinal locations [39].

### Alterations on energy metabolism and production of SCFAs

SCFAs are the primary products of bacterial metabolism of proteins, peptides, complex carbohydrates, and intermediates like lactate and succinate. They are also recognized as beneficial for the host’s physiology and immune system [4, 40]. We observed a significant reduction in lactate and butyrate concentrations during the exposure phase. These findings are consistent with and expand upon our previous study (Fig. S5C). Where exposure of SIHUMIX to BB in a batch culture resulted in a reduction in butyrate but not lactate.

To understand the mechanisms behind the decrease of lactate, we found that abundance of pyruvate kinase (PK), which converts phosphoenolpyruvate into pyruvate, was significantly reduced (Log_2_FC = −0.4). This enzyme and the product of its reaction feed into various downstream pathways [41], making it a critical metabolic intersection whose inhibition can severely impact metabolism [42]. The precise mechanisms by which BB affects the expression of the gene encoding PK remain unknown. However, based on the observed reduction in subsequent energy metabolism proteins, we speculate that BB may lead to decreased PK activity. Other triarylmethane dyes, such as malachite green, have been shown to interact with proteins, altering their structures by loosening folding, reducing β-sheet content, increasing α-helix formation, and ultimately diminishing enzyme activity. [43]. In our study, we did not measure BB within the cytoplasm, and further investigation into the intracellular presence of the colorant should be conducted.

Reduction in butyrate production may be linked to the decreased relative abundance of *C. butyricum*. During the establishment of the SIHUMIx model, its predecessor, SIHUMI (which contained seven bacterial species), exhibited low butyrate production, which increased upon the inclusion of *C. butyricum* [19]. Additionally, four proteins involved in butyrate metabolism were observed to decrease. While the exact mechanisms remain unclear, this reduction may be the result of a cascade of events triggered by impaired metabolism, potentially due to a decrease in PK activity, or by the decline in the relative abundance of *C. butyricum*.

### Effects on pathways, untargeted metabolome and correlations

Functional analyses of food additives’ effects on microbiota models have primarily focused on evaluating the impact of colorants on community composition using metagenomic tools [44, 45]. These approaches describe the changes in bacteria relative abundance and infer their function through predictive tools [46, 47]. In contrast, our metaproteomic approach complements metagenomic studies by describing structural and functional changes, offering a more comprehensive understanding of these effects.

The analysis of proteins assigned to KEGG metabolic pathways revealed substantial changes primarily between the stabilization and exposure stages. Of the 79 identified pathways, 19 demonstrated significant changes that should be considered during analysis. Contrasting with our previous study (Fig. S5B), we found two similarities in affected pathways: terpenoid backbone biosynthesis was downregulated, and the drug metabolism-other enzymes pathway was upregulated after exposure in batch mode. All other pathways identified in this study were not affected in our previous research.

Metabolic pathways related to compound expulsion and antibiotic resistance notably increased during exposure. These results suggest that SIHUMIx entered an adaptive response phase aimed at mitigating the effects of the colorant, potentially through mechanisms of compound expulsion and antimicrobial resistance [48]. This is evidenced by the increase in drug metabolism and protein export systems pathways, as well as significative changes (KW-test, *P* < .05) in proteins involved in cell wall construction and strengthening, such as penicillin-binding proteins, D-ala-D-ala ligases, and MurF fusion proteins (Fig. S6).

Conversely, metabolic pathways such as carbon metabolism, and several amino acid metabolism pathways were negatively affected during the exposure phase. This reduction likely reflects a shift in metabolic priorities, possibly due to stress-induced resource allocation from energy production and biosynthesis to protective mechanisms [49]. The observed decrease in SCFAs, along with enzymes involved in energy metabolism and the reduction in the concentration of seven amino acids, provides an additional layer where we can correlate proteomic impact with metabolic pathway endpoints.

Correlation analysis of metabolic pathways in specific SIHUMIx species provided further insights into their adaptive responses throughout the experimental period. The strains that showed correlations with pathways during the exposure phase (*B. producta, E. coli,* and *C. butyricum*) experienced a significant decrease in abundance when the chemical was added (Fig. 3B). However, during the duration of the exposure phase (Days 7 to 13), their relative abundances had no significant changes. Suggesting that their metabolism adapted to a constant perturbation independent of their abundance.

For *B. producta* we observed a positive correlation between drug metabolism pathway and exposure day (*P* = .55, *P*_adj_ = .002), suggesting its involvement in detoxification processes [50]. For *E. coli*, a significant negative correlation between exposure days and taurine and hypotaurine metabolism (*P* = −.76, *P*_adj_ = .0001) could indicate alterations in amino acid utilization under BB-induced stress [51]. Specifically, the reduction of taurine metabolism may cause negative effects on microbiota homeostasis, as taurine uptake has a positive effect on SCFA production and reduces the abundance of harmful bacteria [52]. The significant correlation between the phosphotransferase system (PTS) and exposure days (*P* = −.57, *P*_adj_ = .0015) observed in *C. butyricum* implies a potential reduction in overall metabolic activity or a shift towards resource conservation under stress conditions [53]. This system, found only in bacteria, is responsible for regulating various cellular processes, such as carbon metabolism, biofilm formation, and chemotaxis [54].

### Recovery of SIHUMIx after removal of brilliant blue

Significant alterations were observed during exposure phase, and after dye removal the community did not return to its pre-exposure profiles. The type of disturbance influences bacterial community responses and recovery, and can be classified as short-term pulses or sustained presses [61]. Experimental and observational studies have shown that communities exposed to press-type disturbances exhibit greater sensitivity than those experiencing pulse-type disturbances, and when evaluating bacterial community recovery, a larger proportion of communities affected by pulse disturbances successfully recover, compared to those subjected to press disturbances [55].

Previous research has demonstrated that SIHUMIx is capable of returning to its pre-exposure state following pulse disturbances, such as a 24-hour pH change [22], and can resist changes from press disturbances, like a 5-day alteration in transit time [21]. However, in the case of pulse exposure to BB, the community did not return to its pre-exposure state after a 4-day recovery period. Suggesting a potential shift to an alternative stable state, where post-exposure structural and functional conditions may persist [56].

*In vitro* studies with other models have shown that, after chemical press-like perturbations and a 14-day recovery period, some aspects, such as relative abundances and diversity indices, may return to pre-exposure levels. However, functional factors such as metabolite production remain affected [57], indicating that additional measures are necessary to restore functionality beyond time alone. Removing the stressor does not guarantee system recovery; factors such as adding of probiotics, and supplementation are required to restore pre-exposure functional and diversity levels [58]. Future research is recommended to compare the effects of longer recovery times, addition of probiotic substances, medium supplements, and re-inoculation of the SIHUMIX strains.

## Considerations and conclusion

The observed effects suggest that BB may negatively impact human microbiota. While this chemical poses no genotoxic, carcinogenic, and reproductive risk to humans, it is not metabolized or absorbed by the body [59, 60], and approximately 96% is excreted in faecal matter [14, 15]. This indicates a high level of exposure of the microbiota to this compound, and the interactions between them have not been well studied so far.

The experimental design of our study aimed to replicate the upper limit of concentration allowed by the European Union. The concentration of 0.168 mg/ml was a scaled proportion of the maximum ADI allowed of 6 mg/kg bw/day (see Methods section). Under these conditions, we observed significant disruptions in the structural and functional integrity of SIHUMIx. Comparing the allowed levels of BB in regions with less stringent regulations, such as the United States, where the ADI is 12.5 mg/kg body weight per day [61] and considering that BB is available to the public in a wide range of products, including soft drinks, confectionery [62], electronic cigarettes [63] and is even proposed for tracking intestinal transit [64] we estimate that the level of consumer exposure is high. Given this extensive exposure, it is crucial to re-evaluate the safety of this compound, with particular attention to its effects on the microbiota and overall host health.

Our study has limitations. While significant effects were observed, the in vitro model used does not fully capture the diversity of faecal samples. Nonetheless, this is offset by the ability to culture bacteria and annotate proteins for species-level functional assignment. Additionally, the model does not account for host influences or complex gastrointestinal interactions. The fate of BB remains partly unknown: 73% was quantified in the supernatant, and no detectable transformation products suggest that SIHUMIx bacteria cannot metabolize it. The coloration in pellets indicates the remaining dye likely adhered to bacterial cells and protein components of the CIM medium. For future studies, we recommend using equations by Lessar-Lord et al. [65], which assess treatment chemical concentrations in bio fermenter setups considering microbial metabolism and dilution effects.

In this study, we observed structural and functional changes in a simplified gut microbiota model using metaproteomic and metabolomic analyses. These changes included the disruption bacterial species, a reduction in lactate and butyrate production, the activation of stress response pathways, and the inhibition of specific metabolic routes to conserve resources during stress. Our objective was to identify the specific impacts on the microbiota and assess whether the community could revert to its pre-exposure state after removing of the food colorant BB. This approach offers valuable insights into the chemical-microbiota interactions and highlights the potential for including this model in regulatory chemical risk assessments.

## Supplementary Material

Supplementary_tables_and_figures_ycaf050

Supplementary_methods_ycaf050

## Data Availability

The mass spectrometry proteomics data have been deposited to the ProteomeXchange Consortium via the PRIDE partner repository [66] with the dataset identifier PXD055767.
