# Supplementary material for: Food colorant brilliant blue causes persistent functional and structural changes in an in vitro simplified microbiota model system
Source: ISME Commun. 2025 Mar 22;5(1):ycaf050. doi: 10.1093/ismeco/ycaf050 (PMC11977461; doi:10.1093/ismeco/ycaf050)
Supplement: Supplementary_tables_and_figures_ycaf050 [file supplementary_tables_and_figures_ycaf050.pdf]

Table S1: Complex intestinal medium (CIM) composition

Ingredient	Quantity [g/L]	Supplier
Arabinogalactan (larch wood)	2	Sigma-Aldrich
Bile Acids sodium salt	0.5	Sigma-Aldrich
Calcium chloride x 2 H_2O	0.01	Merck
Casein peptone (pancreatic)	4.3	Roth
Di-Potassium hydrogen phosphate	0.04	Roth
Hemin (bovine)	0.005	Sigma-Aldrich
Inulin	1	Serva
L-cysteine hydrochloride	0.5	Biochemica
Magnesium sulfate	0.01	Roth
Menadione	0.001	Sigma-Aldrich
Mucin (porcine gastric Type II)	4	Sigma-Aldrich
Pectin, citrus peel	2	Sigma-Aldrich
Potassium di-hydrogen phosphate	0.04	Roth
Sodium chloride	0.72	Roth
Sodium hydrogen carbonate	2	Roth
Starch, wheat	5	Roth
Xylo-oligosaccharide (corn)	2	Roth
Yeast extract	2	Chemsolut

Table S2: Isolation windows for the DIA LC-MS/MS measurements. 48 scanning events, each resulting from equally spaced windows of 24 m/z with a 1 m/z overlap

Window	m/z range	Window	m/z range
1	349.408-374.420	25	925.670-950.682
2	373.419-398.430	26	949.681-974.692
3	397.430-422.441	27	973.692-998.703
4	421.441-446.452	28	997.703-1022.714
5	445.452-470.463	29	1021.714-1046.725
6	469.463-494.474	30	1045.725-1070.736
7	493.474-518.485	31	1069.736-1094.747
8	517.485-542.496	32	1093.747-1118.758
9	541.496-566.507	33	1117.757-1142.769
10	565.506-590.518	34	1141.768-1166.780
11	589.517-614.529	35	1165.779-1190.791
12	613.528-638.540	36	1189.790-1214.802
13	637.539-662.551	37	1213.801-1238.812
14	661.550-686.561	38	1237.812-1262.823
15	685.561-710.572	39	1261.823-1286.834
16	709.572-734.583	40	1285.834-1310.845
17	733.583-758.594	41	1309.845-1334.856
18	757.594-782.605	42	1333.856-1358.867
19	781.605-806.616	43	1357.867-1382.878
20	805.616-830.627	44	1381.878-1406.889
21	829.626-854.638	45	1405.888-1430.900
22	853.637-878.649	46	1429.899-1454.911
23	877.648-902.660	47	1453.910-1478.922
24	901.659-926.671	48	1477.921-1502.933

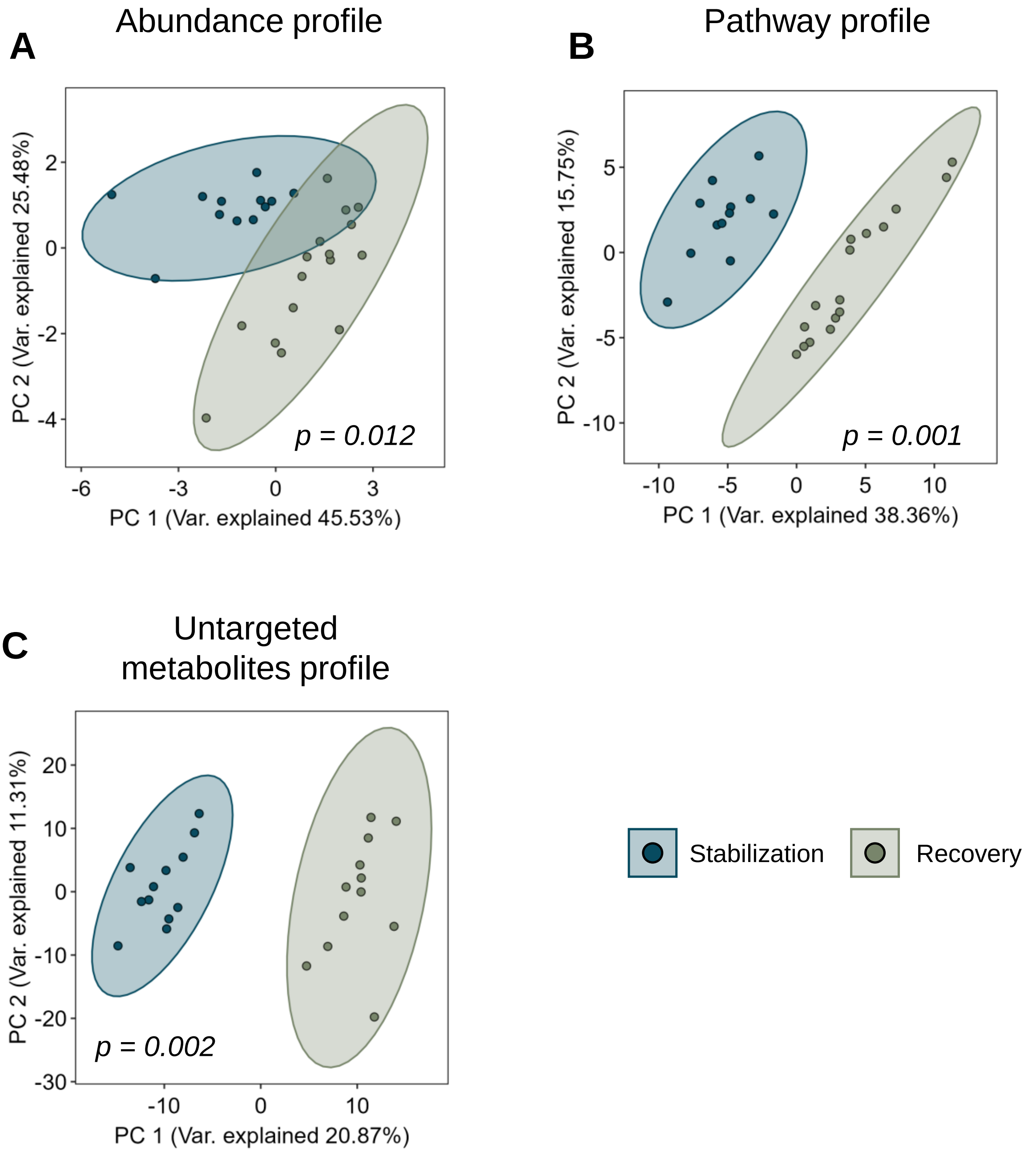

Figure S1. Principal component analysis comparing stabilization to recovery stages of (A) species profile, (B) pathway profile, and (C) untargeted metabolite profile. Significance was calculated by PERMANOVA.

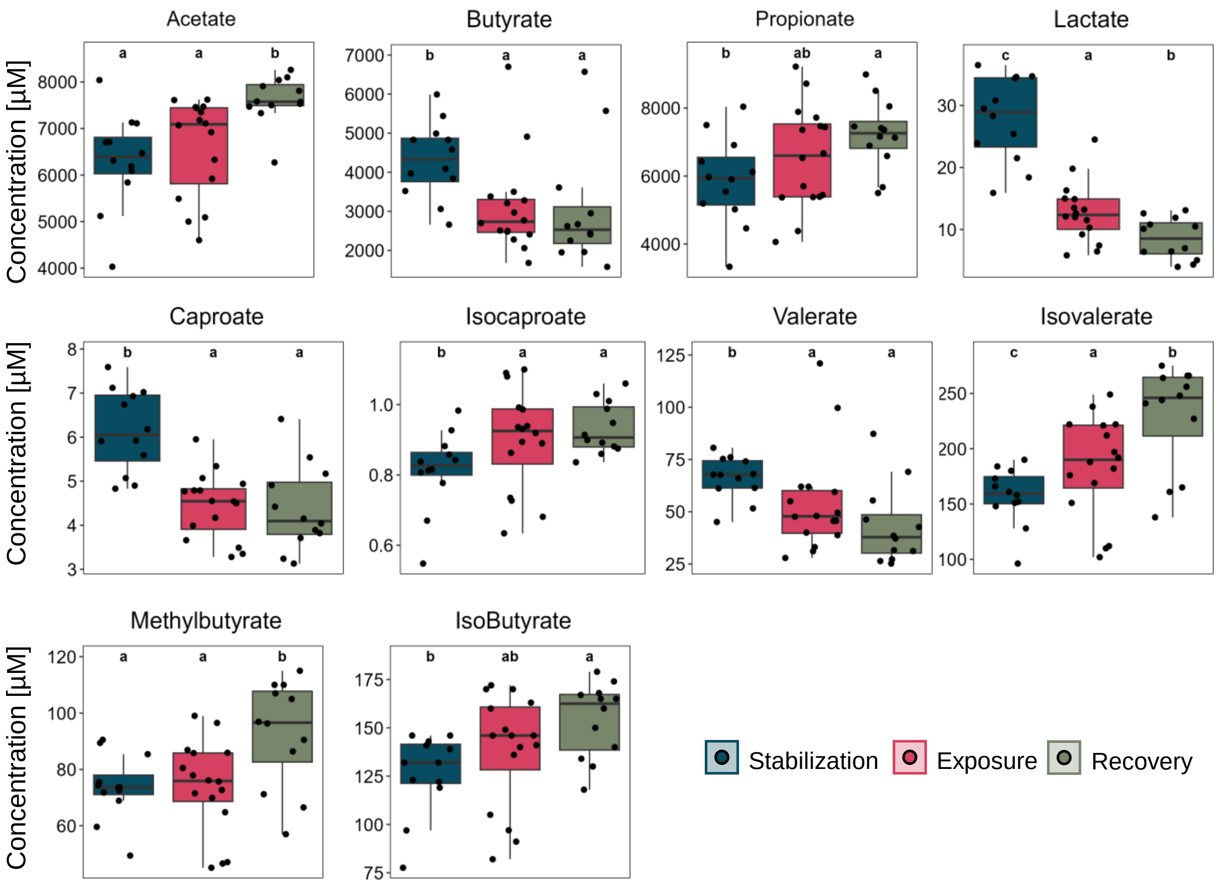

Figure S2. Concentration of SCFAs detected. The concentrations of the ten SCFAs detected in the supernatants of SIHUMIx were measured during the various stages of the experiment. Significant differences, indicated by letters above the plots, were determined using post-hoc tests calculated by the Kruskal-Wallis and Dunn's tests ($p<0.05$). Points represent samples taken from the four bioreactors during each cultivation phase

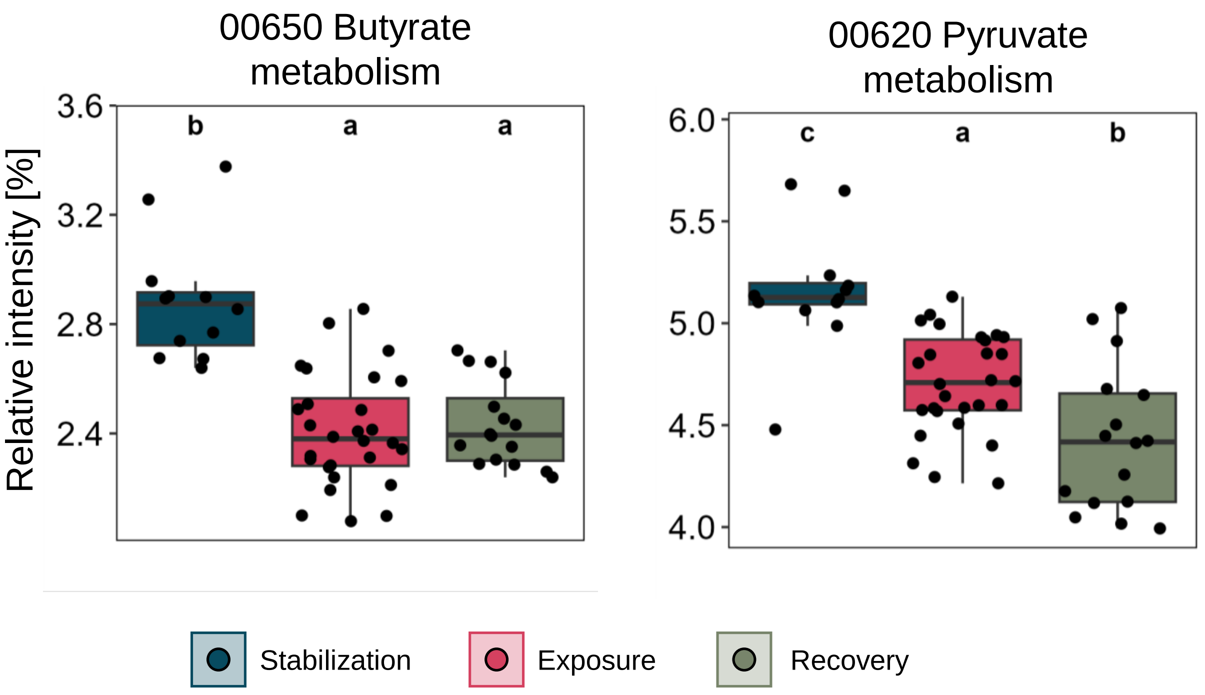

Figure S3. Relative intensity of butyrate and pyruvate metabolism pathways. The relative abundance of the butyrate and pyruvate metabolism pathways was measured before, during, and after exposure to BB. Significant differences, indicated by letters above the plots, were determined using post-hoc tests calculated by the Kruskal-Wallis and Dunn's tests ($p<0.05$). Points represent samples taken from the four bioreactors during each cultivation phase

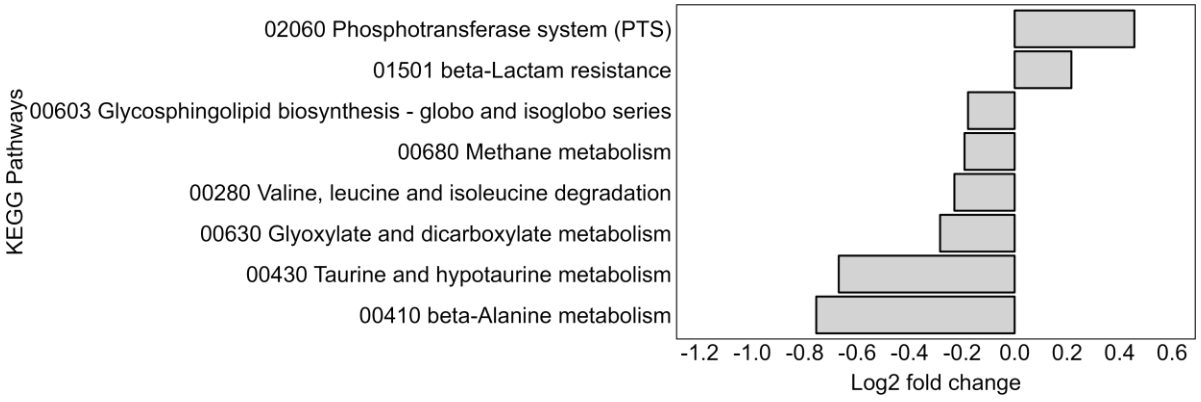

Figure S4. Relevant pathways during the recovery phase. Pathways of interest were identified by comparing the exposure and recovery phases. These pathways were filtered based on a threshold of $Log_2FC=\pm0.175$ and statistical significance, as calculated by the Kruskal-Wallis test ($p<0.05$)

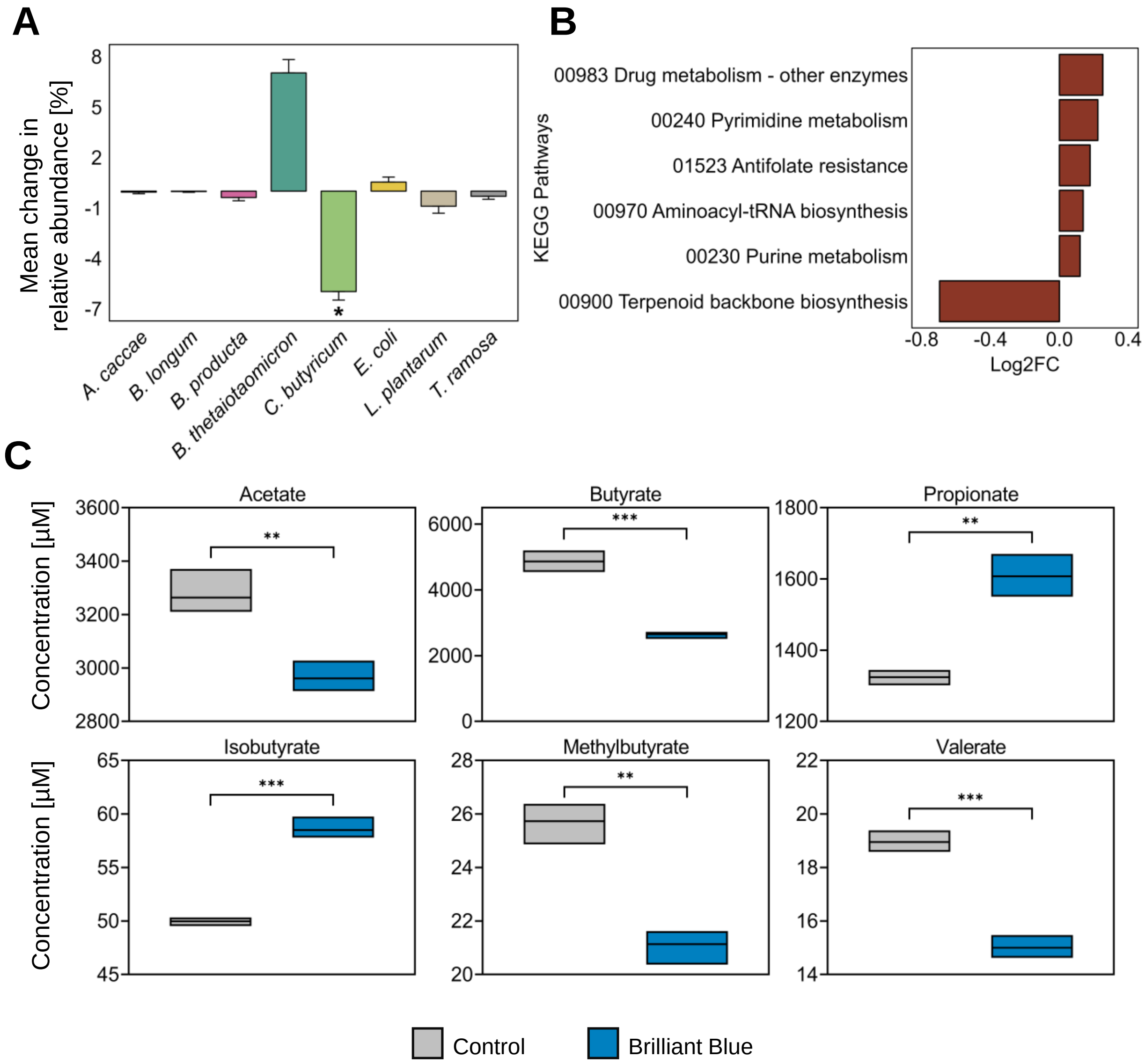

Figure S5. Impact of acute exposure to brilliant blue. Analysis of the structure of SIHUMIx during the stages of the experiment. (**A**) Change in the relative abundance of each SIHUMIx species compared to the control. (**B**) Pathways of interest identified by comparing the BB treated culture to the control. These pathways were filtered based on a threshold of $Log_2FC=\pm0.1$ and statistical significance ($p<0.05$). (**C**) Concentrations of the eight SCFAs affected after acute exposure of SIHUMIx to BB compared to the control. Significant differences, indicated by letters above the plots, were determined using post-hoc tests calculated by the Kruskal-Wallis and Dunn's tests ($p<0.05$).

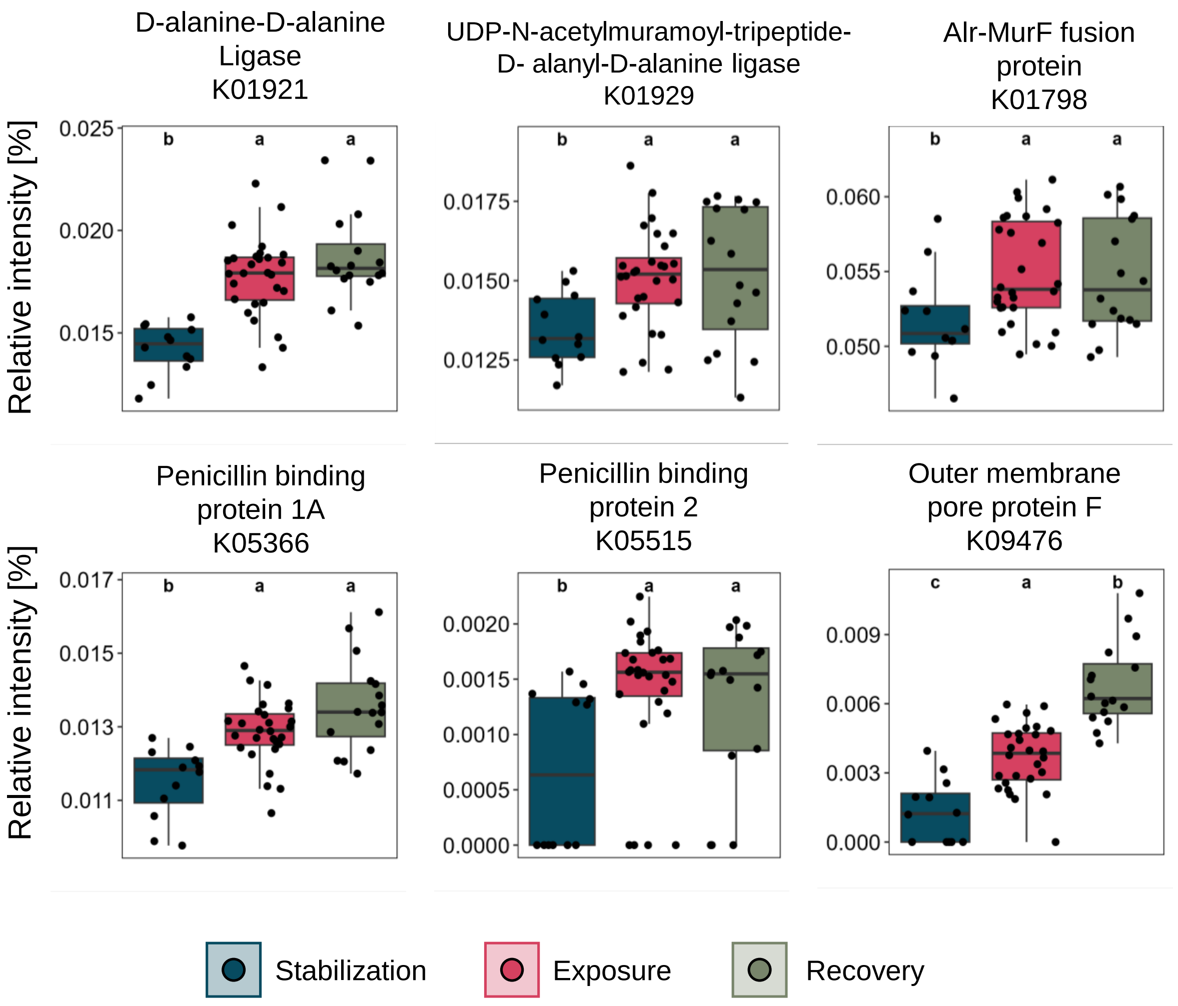

Figure S6. Relative intensities of proteins with structural function. The relative abundance of proteins involved in cell wall construction, reinforcement, and compound transport was measured across the three experimental stages of exposure to Brilliant Blue. Significant differences, indicated by letters above the plots, were determined using post-hoc tests calculated by the Kruskal-Wallis and Dunn's tests ($p<0.05$). Points represent samples taken from the four bioreactors during each cultivation phase

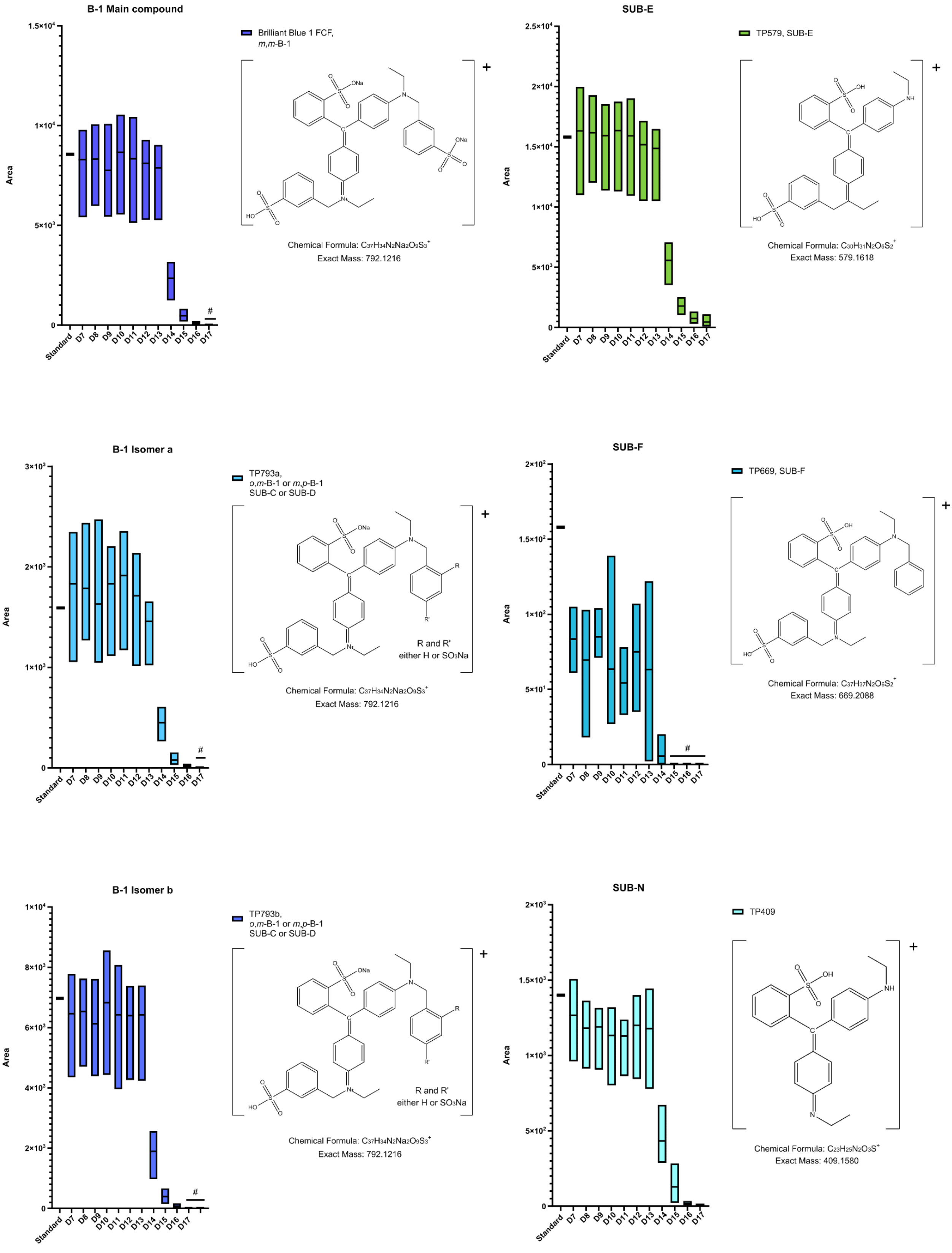

Figure S7. Areas of BB and its subsidiary colours (SUB-C to SUB-F and SUB-N) in standard compared to areas in bioreactor (n=4) from day 7 to day 17. Data presented as interleaved low-high floating bar plot (line at mean) for each compound with names from literature in the legend and structure of the detected molecule ion (m: meta, o: ortho p: para). Exposure time from day 7 to day 13 (D7 to D13) and phase out from day 14 to day 17 (D14 to D17). Measurements under limit of detection are marked with #.
