# Supplementary material for: Food colorant brilliant blue causes persistent functional and structural changes in an in vitro simplified microbiota model system
Source: ISME Commun. 2025 Mar 22;5(1):ycaf050. doi: 10.1093/ismeco/ycaf050 (PMC11977461; doi:10.1093/ismeco/ycaf050)
Supplement: Supplementary_methods_ycaf050 [file supplementary_methods_ycaf050.docx]

## Considerations about the Simplified Human Microbiota Model (SIHUMIx) – From section 2.1

This microbiota model, developed in murine systems using bacteria from the human gastrointestinal tract, captures the core phyla and key metabolic functionalities of the human microbiota. It presents several advantages: it demonstrates functional capacities in murine models comparable to those observed in individuals with a native microbiota, can be successfully transferred to in vitro systems, and consists of species that are comprehensively characterized at both genomic and proteomic levels.

## Sample preparation for metaproteomic analysis - Single-Pot Solid-Phase-enhanced Sample Preparation (SP3) – From section 2.3

30 μg of protein was added to a 1.5 mL Eppendorf tube (Hamburg, Germany), and the sample volume was adjusted to 200 μL with 100 mM Tetraethylammonium bromide buffer (TEAB). The protein sample underwent reduction (2.5 mM Tris (2-carboxyethyl) phosphine hydro-chloride, TCEP, with a 1-hour incubation at 55°C) and alkylation (9 mM Iodoacetamide, IAA, for 30 minutes at room temperature in the dark). Subsequent to alkylation, 260 µL of Acetonitrile (ACN) was added to the sample.

The samples were combined with 50 µg of Sera-Mag Carboxylate-Modified Magnetic Beads & SpeedBeads (10 mg/mL, Cytiva, Marlborough, Massachusetts) and underwent an incubation period at room temperature for 8 minutes. Following a 2-minute rest on a magnetic rack, the supernatant was removed, and the beads underwent two rinses with 200 μL of 70% ethanol and one rinse with 200 μL ACN. After all rinsing steps the supernatant was removed after 2 min incubation on the magnetic rack.

The air-dried beads were reconstituted in 5 μL of a 100 mM TEAB digestion solution containing 0.6 μg of Trypsin/Lys-C Mix, Mass Spec Grade (Promega, Madison, United States), and were allowed to incubate for 16 hours at 37°C. Subsequent to digestion, 150 μL of ACN was added to each sample to achieve a final concentration of > 95% ACN. The samples underwent an 8-minute incubation at room temperature before being placed on a magnetic rack for 2 minutes. Following this, the supernatant was discarded, and the beads were rinsed with 200 μL of 100% ACN; the supernatant was removed after 2 minutes on the magnetic rack.

The rinsed beads were reconstituted in 50 μL of 2% DMSO, sonicated for 1 minute, and incubated for 2 minutes on the magnetic rack to recover eluted peptides in the supernatant. A second elution under the same conditions, without sonication, was performed, resulting in a total of 100 μL of eluted peptides. The peptides were vacuum-dried and resuspended in 30 μL of 0.1% Formic Acid (FA), then subjected to centrifugation at 20,000 g for 10 minutes to eliminate any beads that could obstruct the analytical column. Finally, 25 μL of peptides were transferred from the tubes to LC-MS vials and stored at -80°C until spectrometric measurements.

## LC-MS/MS measurements - From section 2.4

For each nanoLC-MS measurement, 1 microgram (μg) of peptides was injected into a Vanquish Neo nanoHPLC system by Thermo Fisher Scientific. Initial trapping occurred on a C18-reverse phase trapping column (Acclaim PepMapTM 100, 75 μm × 2 cm, particle size 3 μm, nanoViper, Thermo Fisher Scientific), followed by subsequent separation on a C18-reverse phase analytical column (Double nanoViper™ PepMap™ Neo, 75 μm × 150 mm, particle size 2 μm, Thermo Fisher Scientific). The separation utilized a two-step gradient employing mobile phases A (0.01% formic acid in H2O) and B (80% acetonitrile in H2O with 0.01% formic acid). During the first 65 minutes, the proportion of mobile phase B increased from 4% to 30%, followed by a subsequent 30-minute period during which the proportion of mobile phase B rose from 30% to 55%. The flow rate was maintained at 300 nL per minute throughout the separation.

The eluted peptides underwent ionization using a Nanospray Flex™ Ion Source (Thermo Fisher Scientific) and were subsequently detected by an Orbitrap Exploris™ 480 mass spectrometer (Thermo Fisher Scientific) operating in data-independent acquisition (DIA) mode. Specific settings for the mass spectrometer were configured as follows: for MS, the scan range was 350–1,500 *m/z*, the resolution was set at 120,000, the automatic gain control (AGC) target was 3,000,000, and an auto maximum injection time. Regarding MS/MS, parameters were set with a resolution of 30,000, AGC at 1,000,000, collision energy at 30%, RF lens at 45%, and maximum injection time set to auto. The DIA window type was automatic with an isolation window of 24 *m/z* and an overlap of 1 *m/z*, spanning a precursor range of 350-1,500 *m/z*, resulting in 48 scanning events (**Table S2**)

## Analysis of Short Chain Fatty Acids (SCFA) - From section 2.7

Samples were mixed with acetonitrile to achieve a final concentration of 50%. For derivatization, 0.5 volumes of 200 mM 3-nitrophenylhydrazine and 0.5 volumes of 120 mM N-(3-dimethylaminopropyl)-N-ethylcarbodiimide hydrochloride in pyridine were added, and the mixture was incubated at 40 °C with shaking at 300 rpm for 30 minutes. The derivatized SCFA solutions were then diluted 1:50 with 10% acetonitrile.

A 10 µL aliquot of the diluted derivatized solution was injected into an RSLC UltiMate 3000® system (Thermo Fisher Scientific) coupled to a QTRAP 5500® mass spectrometer (AB Sciex, Framingham, MA, USA). SCFAs were separated using an Acquity UPLC BEH C18 column (1.7 µm; Waters, Eschborn, Germany) with water (0.01% formic acid) and acetonitrile (0.01% formic acid) as mobile phases. The flow rate was set at 0.35 mL/min, with the column maintained at 40 °C. The elution gradient was: 2 minutes at 15% B, a 15-minute gradient from 15% to 50% B, 1 minute at 100% B, and 3 minutes re-equilibration at 15% B. SCFAs were identified and quantified using a scheduled MRM method with specific transitions for each SCFA. Peak areas were analysed using Analyst® Software (v1.6.2, AB Sciex), and the data were exported.

Metabolite quantification utilized custom R scripts developed in R Studio (v2023.03.0) and calibration curves.

## Untargeted metabolomics – From Section 2.8

Prior to analysis, samples were mixed with five volumes of MeOH: ACN:H_2_0 in a 2:3:1 (v:v:v) ratio, sonicated and centrifuged. 550 µL of the supernatant was transferred and evaporated. Prior to measurements samples were resuspended in 100 µL 0f 0.1% formic acid and 1% ACN in water.

From the resuspended samples, a pooled sample consisting of equal volumes taken from all samples was prepared and spiked with a mix of internal standards. The standards had a final concentration 5.5 µM.

Samples were analysed using a high-performance liquid chromatography (HPLC) system (Vanquish, Thermo fisher Scientific, MA, USA) coupled to a mass spectrometer (Orbitrap IQ-X, Thermo Fisher Scientific, MA, USA), operating in AcquireX mode (Thermo Fisher Scientific, MA, USA). Each sample was run in two separate batches, with the first batch analysed in positive ionization mode and the second batch in negative ionization mode.

For each analysis, 5 µL of the sample was injected into the HPLC and initially trapped on a precolumn (ACQUITY UPLC HSS T3 VanGuard precolumn, 2,1mm X 5mm,186003976, Waters GmbH, Eschborn, Germany). The sample was then separated on an analytical column (ACQUITY UPLC HSS T3 Columns,2.1 mm X 100 mm, 186003539, Waters GmbH, Eschborn, Germany) with a solvent flow rate of 0.3 mL/min at a column temperature of 40°C. The gradient started with 99% solvent A (Milli-Q water with 0.1% formic acid) and 1% solvent B (acetonitrile with 0.1% formic acid) for the first 3 minutes. Solvent B was then linearly increased to 35% by 15 minutes and further to 99% by 20 minutes. The 99% solvent B was maintained for 2 minutes until 22 minutes, after which it was rapidly reduced to 1% by 22.5 minutes and held until the end of the 25-minute run.

Eluting compounds were ionized using an ESI source (Ion-Max NG, Thermo fisher Scientific, MA, USA) either in positive mode (3400 V) or negative mode (-2600 V). The source parameters were set as follows: sheath gas at 40 units, auxiliary gas at 5 units, sweep gas at 1-unit, vaporizing temperature at 400°C, and ion transfer tube temperature at 300°C.

For metabolite identification, samples were analysed using both MS/MS and Full-MS methods. A blank sample containing only solvent A was run first to generate an exclusion list of contaminant features. A pooled sample was then analysed in Full-MS mode to detect features, followed by five MS/MS runs of the pooled sample. In each MS/MS run, previously identified features were excluded from MS2, allowing the fragmentation of unique features and maximizing the number of metabolites identified in subsequent data analysis.

All individual samples were then analysed using the Full-MS method. Quality control (QC) samples, consisting of pooled samples, were measured at the beginning, middle, and end of each batch. Each QC consisted of four pooled samples analysed using the Full-MS method to ensure consistency and reliability throughout the analytical run.

The MS-settings for the MS/MS identification method were: Orbitrap resolution 60,000, quadrupole isolation used, Scan range 50-1000 m/z, Maximum injection time 50 ms, RF Lens 35%, Polarity either positive for positive measured batch or negative for negative measured batch. Filter type: Intensity threshold of 20,000. Dynamic exclusion: Exclude after 1 time, exclusion duration 2.5 s, mass tolerance low 5 ppm and high 5 ppm, exclude isotopes, exclude within cycle. Targeted mass: Mass list type is m/z, time mode is start/End time, include intensity threshold, add mass list targets determined by Xcalibur AcquireX (Thermo fisher Scientific, MA, USA), mass tolerance low 5 ppm and high 5 ppm. MS2 settings were detector Orbitrap, isolation mode Quadrupole, isolation window 1.5 m/z, activation type HCD, collision energy type was stepped, HCD collision energy type was normalized, HCD collision energies were 320%, 30% and 35%, orbitrap resolution 30,000, scan range mode was auto, AGC target was standard, maximum injection time mode was custom with the maximum injection time 50 ms, micro scans were 1, and data type was profile.

For the relative quantification only Full-MS method the MS-settings were: Orbitrap resolution 120,000, Scan range 50-1000 m/z, Maximum injection time 50 ms, RF Lens 35%, Polarity either positive for positive measured batch or negative for negative measured batch, and data type was profile.

Data was collected in *.RAW format and processed using Compound Discoverer^tm^ (Thermo fisher Scientific, MA, USA).

The Compound Discoverer output, containing peak normalized abundances and compound annotations, was filtered to retain only peaks that met the following criteria: an associated MS2 spectrum, a minimum mzCloud best score of 50, a peak rating of at least 3.5 in two or more samples, and a mass deviation of less than 5 ppm from the theoretical compound mass. For compounds annotated to multiple peaks at different retention times, only the peak with the highest normalized abundance across samples was selected. Additionally, normalized peak abundances from peaks annotated to the same compound and occurring within 30 seconds of the peak with the highest abundance were summed with the value of that peak.

## Measurement of Brilliant Blue – From Section 2.9

Prior to analysis, samples were thawed, homogenized, and centrifuged at 10,000 rpm for 10 minutes at 4°C. One sample from each bioreactor was analysed at each time point (n=4). The samples were then diluted with ultrapure water to a final concentration of 168 ng/mL (1:1,000) before chemical analysis was performed using ultraperformance liquid chromatography coupled with time-of-flight mass spectrometry (UPLC-TOF-MS). The analysis was conducted on an ACQUITY UPLC I-Class system (Waters, Milford, MA, USA), equipped with an HSS T3 column (100 mm × 2.1 mm, 1.8 μm), and coupled to a XEVO G2S mass spectrometer (Waters). On the last two days of sampling, the concentration of Brilliant Blue fell below the limit of detection (LOD); therefore, these samples were reanalyzed after a 1:10 dilution.

The mobile phase for the LC system consisted of water with 2 mM ammonium formate and 0.1% formic acid (v/v) as solvent A, and methanol with 2 mM ammonium formate and 0.1% formic acid (v/v) as solvent B. The solvent gradient was as follows: 0 min, 2% B; 12.25 min, 99% B; 15.00 min, 99% B; 15.10 min, 2% B; and 17.00 min, 2% B. The UPLC-TOF-MS was operated in MSe mode with the following parameters: flow rate, 0.45 μL/min; column temperature, 45°C; capillary voltage, 0.7 kV; source temperature, 120°C; desolvation temperature, 600°C; sampling cone voltage, 30 V; source offset, 15 V; cone gas, nitrogen; collision gas, argon; desolvation gas flow, 950 L/h; scan time, 0.15 s; and collision energy, 20–30 eV.

TargetLynx software (Waters, version 4.2) was used for the quantification of analytes through external calibration. Calibration standards were prepared from the same stock solution used in the experiment, ranging from 0.3 ng/mL to 300 ng/mL. For the identification of transformation products, the same data were processed using MarkerLynx XS™ v4.2 software (Waters) to generate feature lists. Peak picking parameters were optimized to search within a retention time window of 0.5–17 minutes (±0.1 min) and a mass range of 50–1,200 Da (±0.01 Da). Chemical formulas were generated with a mass tolerance of 5 ppm, considering the elemental composition of the parent compound. The generated feature lists were compared with suspect compounds reported in the literature. Purity criteria for food colorants of the commission directive 2008/128/EC define educts and the sum of by-products of food colorants formed during synthesis, also called subsidiary coloring matters. Structures of the latter were described for Brilliant Blue in literature and were used as a suspect list (SUB-C to SUB-F and SUB-M).

Five subsidiary colors were detected, with four known and one previously unknown structure. The known structures consisted of the two isomers of Brilliant Blue (SUB-C, SUB-D) and three smaller derivates (SUB-E, SUB-F and new SUB-N). Without commercially available standards, areas were compared between standard before and after addition and no significant changes were observed (**Figure S6**) during exposure from day 7 to day 13 (D7 to D13). For SUB-F, the signal appeared to decrease after addition to the bioreactor. However, given its proximity to the limit of detection, increased variability is expected and no new signals were detected during the bioreactor run to support a possible degradation.
